# Bearing Capacity and Deformation of the Tandem Compound Piles Improved Foundation: A Parametric Study

**DOI:** 10.3390/ma16175737

**Published:** 2023-08-22

**Authors:** Youlin Guo, Xiaocong Cai, Meixiang Gu

**Affiliations:** 1 Hunan Engineering Research Center of Structural Safety and Disaster Prevention for Urban Underground Infrastructure, Hunan City University, Yiyang 413000, China; guoyoulin0708@163.com; 2 School of Civil Engineering, Guangzhou University, Guangzhou 510006, China; 2112116004@e.gzhu.edu.cn

**Keywords:** parametric analysis, tandem compound piles, bearing capacity, discrete element method, finite difference method

## Abstract

The tandem compound piles are a combination of a granular column in the deep section and a concrete pile in the shallow section. This method effectively utilizes the consolidation and densification effects of the granular column, as well as the cementation strength of the concrete material. The granular column acts as a consolidation path, aiding in the densification of the surrounding soil. On the other hand, the concrete pile prevents the bulging deformation that commonly happens in granular columns during field construction. To study the bearing capacity and deformation of the improved foundation with tandem compound piles, a coupled continuum-discrete numerical model was developed in this study. The accuracy of the model was confirmed by comparing its results with experimental measurements. Additionally, a parametric study was conducted, considering three influential factors: (1) cushion thickness and modulus, (2) length, modulus, diameter, and spacing of the tandem compound pile, and (3) soil modulus. The results indicated that reducing the cushion thickness and increasing the cushion modulus allowed the pile to bear more loads. Moreover, increasing the length and modulus of the deep section of the pile reduced deformation and improved the bearing capacity. The pile modulus, however, had a limited effect on enhancing the bearing capacity. It is important to maintain a critical pile spacing of at least twice the pile diameter. Finally, a high modulus of the underlying stratum led to higher vertical and radial stresses in the pile.

## 1. Introduction

The granular column is widely recognized as a cost-effective solution for reinforcing foundations. It has been extensively adopted to enhance the load-bearing capacity of soft soil foundations, including embankments, low-rising structures, and storage tanks [1,2,3,4]. In this technique, crushed gravel typically replaces the soft soil as a pile, which stiffens the bearing capacity, stability, and drainage of the foundation [5,6,7]. However, the efficiency of this method largely depends on the lateral constraints provided by the surrounding soil, which determine the bearing capacity of the composite foundation [8,9,10]. It is crucial that the undrained shear strength of the surrounding soil is not less than 15 kPa [11]. Unfortunately, due to the inadequate lateral confinement provided by the surrounding soil, granular columns often encounter issues such as bulging failure, overall shear failure, and punching failure [12,13,14]. Generally, the primary failure mode is bulging deformation, which typically occurs in shallow soil layers within a depth of one to three times the diameter of the column [15,16]. The environmental friendliness, low cost, and convenient construction are advantages of the granular column [17]. There are various methods to improve its bulging deformation, such as the geosynthetic reinforcement and the concrete pile. The geosynthetic reinforcement can be placed either in the vertical direction as a reinforcement sleeve to constrain the bulging deformation or in the horizontal direction to stabilize the granular material. The concrete pile can be used to replace the granular column in shallow depths to eliminate the bulging deformation.

Geosynthetic materials have garnered attention due to their excellent tensile performance [18] and their efficacy in enhancing granular columns to improve weak foundations [19]. These materials (typically geogrids and geotextiles) are used to vertically encase and horizontally reinforce the granular column, addressing the insufficient lateral confinement of the surrounding soil [20,21,22,23]. Researchers have conducted extensive theoretical and experimental work in this field and have found that the use of geosynthetics effectively improves bearing capacity and deformation [24,25,26]. Cheng et al. [27] conducted a numerical simulation of a floating geogrid-encased stone column to analyze the failure mode and load transfer law of such composite foundations and found that the long column was more prone to generate bulging deformation. Ou Yang et al. [28] investigated the effects of geosynthetic-encased and ordinary stone columns on acceleration amplitude and frequency based on model tests. They found that the former had a narrower crack distribution than the latter. Gniel and Bouazza [29] proposed a new construction method for geogrid-encased stone columns based on laboratory testing and concluded that the overlap could provide an alternative to welding for geogrid-encased stone columns.

Nevertheless, the construction method of geosynthetic-encased stone columns in practical engineering is relatively difficult and controls the construction quality. To further improve working performance, increase economic benefits, and decrease the complexity of construction, a new type of tandem compound piles using concrete-discrete material was developed to reinforce these foundations [30]. The granular column is constructed in the deep section to transfer the vertical loads to deep soils. The concrete pile is constructed in the shallow section to eliminate the bulging deformation. This method effectively utilizes the consolidation and densification effects of the granular column, as well as the cementation strength of the concrete material. Moreover, this technique has the potential to expand the practical application and enrich the theoretical system of composite foundation engineering. However, the lack of information about this new type of tandem compound piles hinders its application and development.

This paper presents a parametric analysis of the new composite foundation using a coupled discrete-continuum numerical method. The numerical model was successfully developed and validated using experimental data. The effects of variations in cushion thickness and modulus, length, modulus, diameter, and spacing of the tandem compound pile and soil modulus on the performance of the tandem compound piles were studied based on the numerical model. The findings of this study can provide valuable data to understand and facilitate the widespread application of the new compound pile.

## 2. Coupled Discrete-Continuum Numerical Method

PFC3D (Particle Flow Code) and FLAC3D (Fast Lagrangian Analysis in the Continua Software Program) have powerful three-dimensional analytical capabilities in modeling discrete materials and continuums, respectively [31]. Moreover, these two programs can realize coupled calculations. The mechanical interaction at the contact boundary between the continuous and discrete domains is defined as discrete-continuum coupling action, and calculation data between different domains are transferred and exchanged via the Socket O/I interface [32]. In continuum calculation, the nodal velocities at the interface are interpolated, and the contact forces from particles are allocated to the interface nodes based on weight functions. Meanwhile, in discrete particle calculation, a particle contact model calculates the interaction between the particles and continua at the interface. To establish a numerical model that satisfies computational accuracy and efficiency, the computation zone of the numerical model was determined through preliminary calculations before creating the model. The coupled continuum-discrete numerical method was a beneficial technique for solving problems in projects composed of complex materials and components. The theory and detailed explanation of this method could refer to the previous studies [33,34,35,36,37,38], and there was no further statement from a concise perspective.

**Figure 1:**
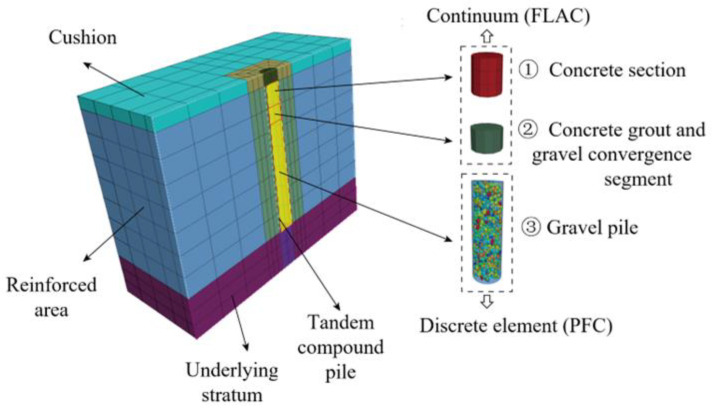
Schematic diagram of a numerical model for the tandem compound pile improved foundation.

The composite foundation comprised the cushion, reinforced area, and underlying stratum. The tandem compound pile was used to reinforce the foundation and concluded two sections: the concrete section (including the concrete grout and gravel convergence segment) and the granular column section. The concrete section and the surrounding soil [39,40] were modeled as a continuous element in FLAC3D, while the particles of the granular column were simulated using PFC3D. Table 1 presents the particle size distribution of the granular column. The composite foundation model had dimensions of 8.6 m × 8.6 m × 19.2 m (length × width × height), with thicknesses of 0.2 m, 16 m, and 3 m for the cushion, reinforced area, and underlying stratum, respectively. The length of the tandem compound pile, which had a diameter of 0.6 m, was 16.0 m, with the concrete section and granular column section being 4.0 m and 12.0 m, respectively. According to the actual condition, only the ground surface was free, and the rest of the surface was fixed with normal displacement as the boundary condition. The loading rate was controlled at 0.0005 m/s to ensure reasonable model calculation. Each time step was 4.5 × 10^−7^ mm/step, and at least 150,000 steps were necessary to experience 1 mm displacement. The ratio of maximum unbalance force to contact force was set as 5‰ to ensure the effective transfer of force between the particle and continuum and to eliminate the impact of unbalance force. Figure 1 presents the schematic diagram of the numerical model for the tandem compound pile improved foundation. The cushion layer, reinforced area, and underlying stratum were uniformly divided into 2, 32, and 6 segments, respectively, in the depth direction. The concrete section and concrete grout and gravel convergence section were divided into 5 and 3 segments, respectively, in the depth direction. In the other two directions, the elements (soil) were all divided into 18 segments unevenly, where the area approaching and intersecting the pile was densified. The pile in the radial direction was divided into three segments. Meanwhile, the particles were generated in the gravel pile section. The numerical model comprised 59,876 continuous elements in FLAC3D and 4891 discrete particles in PFC3D.

**Figure 2:**
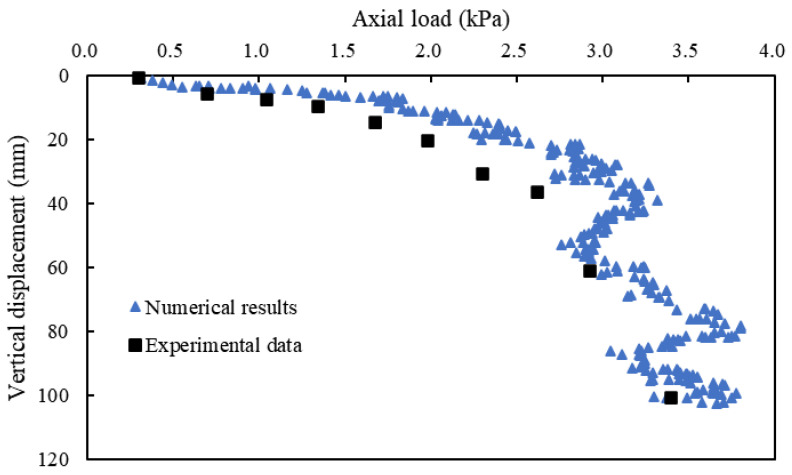
Comparison between the numerical results and laboratory tests [41].

This study aims to investigate the impact of each pile section (e.g., the concrete section and the granular column section) on the bearing capacity and deformation of composite foundations. To ensure the validity and reliability of the coupled numerical model, the experimental results presented by Gu et al. [41] on ordinary stone column-improved soft clay were referenced. A comparison was made between the numerical results and laboratory tests, as shown in Figure 2. The curves from the numerical calculations matched well with the experimental data, affirming the reliability of the numerical model for analyzing the new type of pile composite foundation. The properties of the tandem compound pile and soil were determined based on the validated model. The properties of the tandem compound pile at different pile sections used in the numerical model are shown in Table 2. The foundation soil included the cushion, reinforced area, and underlying stratum. The properties of the foundation soil are presented in Table 3.

## 3. Parametric Study

### 3.1. Cushion Thickness and Modulus

The bearing capacity of the tandem compound pile improved foundation was dependent on the cushion thickness and modulus. In the numerical model, the cushion thickness and modulus were adjusted, and other parameters were kept constant. Five models with varying cushion thicknesses (i.e., t_1_ = 0, t_2_ = 100 mm, t_3_ = 200 mm, t_4_ = 300 mm, and t_5_ = 400 mm) and four models with different cushion moduli (i.e., $E_{1}^{c}$ = 30 MPa, $E_{2}^{c}$ = 50 MPa, $E_{3}^{c}$ = 90 MPa, and $E_{4}^{c}$ = 150 MPa) were established. These numerical models were used to study the effect of cushion thickness and moduli on the stress and deformation characteristics of the pile and soil.

### 3.2. Pile Parameter

**Figure 3:**
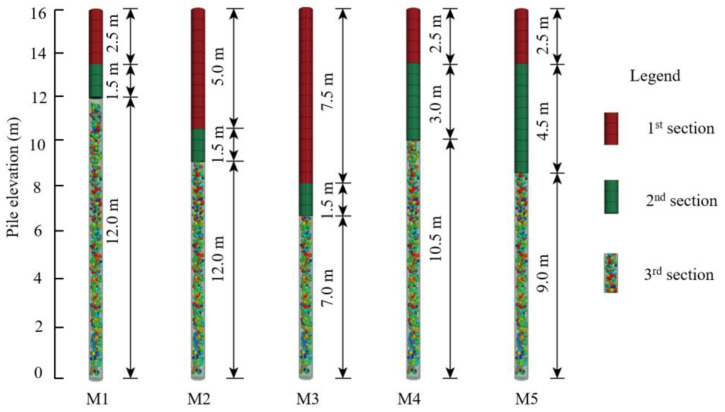
Schematic diagram of pile sections with different lengths.

The impact of pile layout and dimensions on the performance of the tandem compound pile improved foundation was assessed by varying the pile length, diameter, and spacing. The pile length was set at 16 m. For this purpose, five models (M1–M5) were established to analyze the effect of the length of different pile sections on the composite foundation, as shown in Figure 3. Furthermore, four models (M10–M13) and four models (M14–M17) were created to investigate the effect of pile diameter and spacing, respectively, on the composite foundation. The concrete strength of the concrete pile played a crucial role owing to the bulging deformation of the granular column in the shallow soil. To explore the effect of concrete strength on the tandem compound pile improved foundation, this paper constructed four models (M6–M9) with different concrete strengths. The pile parameters are summarized in Table 4, where the elastic modulus for C15, C20, C30, and C40 were 2.20, 2.55, 3.00, and 3.25 MPa, respectively. The lengths of each section in M6–M17 were consistent with the values in M1.

### 3.3. Soil Modulus

The different properties of foundation soils can significantly affect the bearing capacity and deformation characteristics. It is essential to analyze the effect of soil modulus in different soils on the bearing capacity and deformation of the tandem compound pile improved foundation. Four models corresponding to different moduli of reinforced soil (i.e., $E_{1}^{r}$ = 10 MPa, $E_{2}^{r}$ = 20 MPa, $E_{3}^{r}$ = 30 MPa, and $E_{4}^{r}$ = 40 MPa) and four models corresponding to different moduli of underlying stratum (i.e., $E_{1}^{u}$ = 8 MPa, $E_{2}^{u}$ = 16 MPa, $E_{3}^{u}$ = 24 MPa, and $E_{4}^{u}$ = 322 MPa) were established to investigate the aforementioned issues.

## 4. Results and Discussion

### 4.1. Effect of Cushion Thickness and Modulus

**Figure 4:**
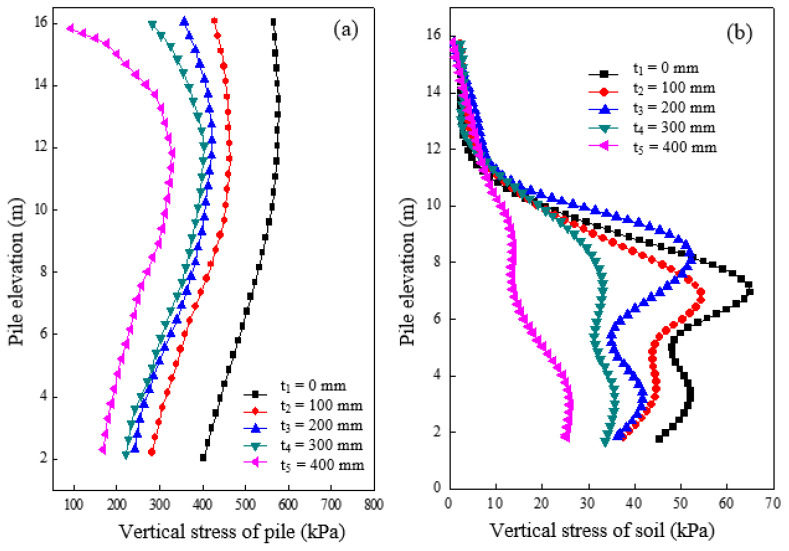
Variation of vertical stress under different cushion thicknesses in (**a**) pile and (**b**) soil.

The changes in vertical stress of both pile and soil caused by different cushion thicknesses after loading are shown in Figure 4a,b. Distinct variations in the vertical stress of the pile can be observed according to the thickness of the cushion in Figure 4a. The pile was subjected to a significant load, and its vertical stress decreased with a decrease in elevation when no cushion was set. However, the vertical stress of the pile reduced substantially with a cushion place, indicating an equalizing effect on the load, which became more pronounced with increasing cushion thickness. Figure 4b shows the vertical stress changes in the soil around the pile at a distance of 0.15 m for varied cushion thicknesses. The results showed that the vertical stress of the soil varied considerably with elevation for different cushion thicknesses. For instance, at a thickness of 400 mm, the vertical stress of soil in the third section resembled the gravitational distribution of soil, while the vertical stress of soil in the first and second sections (elevation 12–16 m) underwent inconspicuous changes. The effect of vertical load and cushion thickness on the vertical stress of soil around the first and second sections was relatively small compared to that around the third section, where it was significant, particularly with lower cushion thickness. It can be concluded that a reasonable cushion thickness is crucial for enhancing the load-carrying capacity and deformation of both pile and soil.

**Figure 5:**
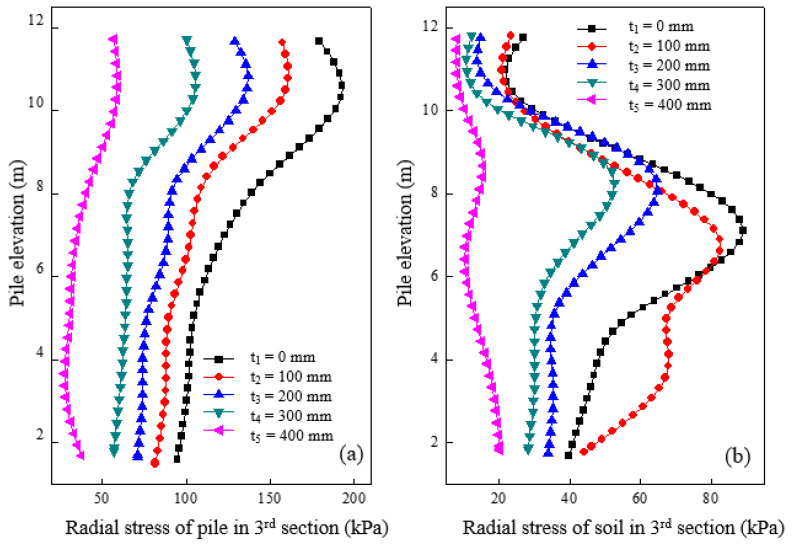
The radial stress variation of granular column section under varying cushion thicknesses after loading in (**a**) pile and (**b**) soil.

Figure 5a shows the radial stress variation of the granular column section under varying cushion thicknesses after loading. The radial stress of the granular column peaked at around 2 m from the top of the granular column section, corresponding to an elevation of 10 m at different cushion thicknesses. The radial stress was higher when the cushion was thicker, or the column was shallower. Figure 5b shows the radial stress changes in soil around the granular column at a distance of 0.15 m from the pile surface at different cushion thicknesses. Radial stress varied significantly at different elevations, with a relatively uniform distribution for a cushion thickness of 400 mm. However, with decreasing cushion thickness, the radial stress of the soil significantly increased, especially below 10 m of elevation. High radial stress indicated more significant bulging deformation around the top of the granular column with a thinner cushion. Consequently, a thicker cushion is preferable to distribute the stress between piles and soil, thereby reducing bulging deformation.

**Figure 6:**
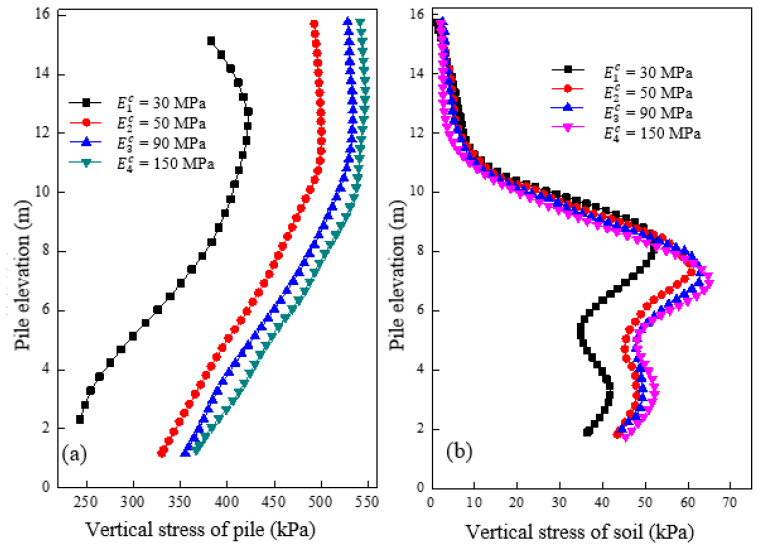
Variation of vertical stress under different cushion modulus in (**a**) pile and (**b**) soil.

Figure 6a shows the distribution of vertical stress along the pile at different cushion moduli after loading. The vertical stress of the pile increased with the increase of cushion modulus. This led to an apparent stress concentration phenomenon at the top of the pile, where a greater load is carried. For instance, the vertical stress at the top of the pile increases by 1.57 times when the cushion modulus increases from 30 MPa to 150 MPa. However, increasing the cushion modulus from 90 MPa to 150 MPa caused only a slight 5% increase in the vertical stress, indicating that the impact of incrementally increasing cushion modulus became less significant on stress adjustment. Figure 6b shows that increasing cushion modulus significantly raises the vertical stress of soil in the granular column section (e.g., elevation of 0–8 m), while the effect on the concrete pile section (e.g., elevation of 8–16 m) is marginal. Any increase of cushion modulus from 90 MPa to 150 MPa will result in a small change in the vertical stress of soil around the granular column section, implying that the vertical stress variation of the soil around this section is similar to that in the granular column section.

**Figure 7:**
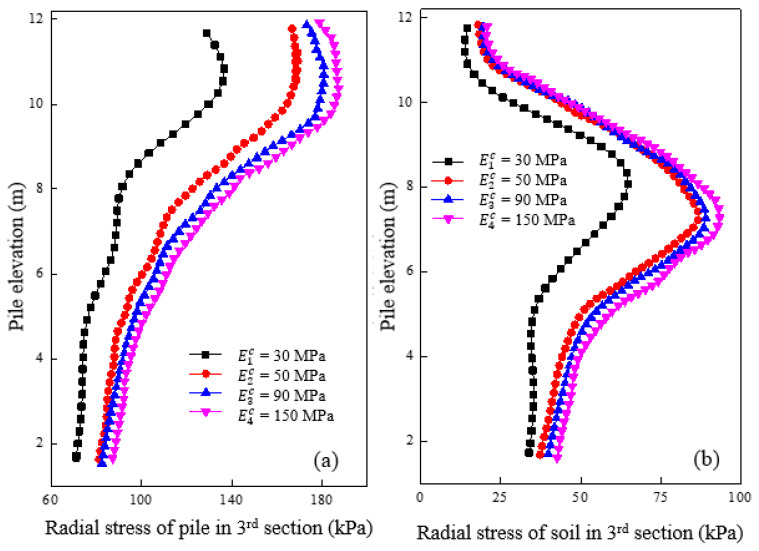
Variation of radial stress under different cushion modulus in the granular column section in (**a**) pile and (**b**) soil.

Figure 7a shows the radial stress variation of the granular column section with different cushion moduli after loading. The radial stress of the granular column section increased gradually with the increase of cushion modulus. However, the influence of the cushion modulus on the radial stress of the granular column section was insignificant until the modulus increased to a certain extent. The radial stress of the granular column increased by 12.4% and 46.0%, respectively, with the cushion modulus increasing from 50 MPa to 90 MPa and 30 MPa to 50 MPa for an elevation of 10 m. Figure 7b shows the radial stress variation curve of the soil at 0.15 m from the pile side with different cushion moduli after loading. The graph indicates that an increase in the cushion modulus also increased the radial stress on the soil around the granular column section. When the cushion modulus increased, the load-carrying capacity of the pile increased, and the load transmitted to the granular column section comparatively increased. This result caused significant bulging deformation and radial stress around the granular column section. The radial stress of the soil increased by 1.5% and 46.4%, respectively, as the cushion modulus increased from 30 MPa to 50 MPa and 50 MPa to 90 MPa, for an elevation of 7 m. A critical cushion modulus value of 50 MPa was significant, leading to increased radial and vertical stresses of both the pile and soil.

### 4.2. Effect of Pile Parameter

**Figure 8:**
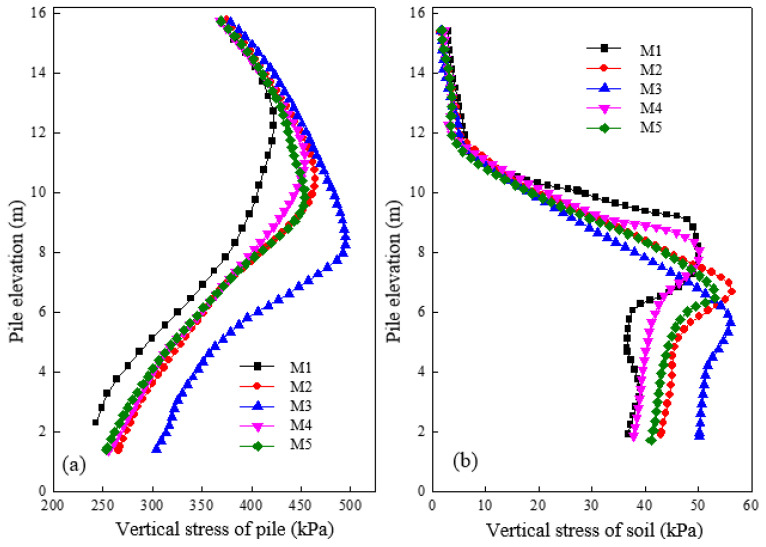
Variation of vertical stress under different lengths of pile section in (**a**) pile and (**b**) soil.

Figure 8a shows the variation of vertical stress for a pile with different lengths of concrete pile after loading. Comparing results from different models (M1, M2, M3) with those having an increased length in the concrete pile section, we can observe a proportional increase in negative friction, resulting in the plane of equal settlement gradually moving deeper. Conversely, in M1, M4, and M5, results show that increasing length in the second section had a minimal impact on the pile’s vertical stress. The length increase in the concrete pile section significantly increased the pile’s vertical stress and bearing capacity, while the increase in the second section had only a slight effect on the vertical stress and bearing capacity. Figure 8b shows the variation of vertical stress in the soil at a distance of 0.15 m from the pile surface under different lengths of pile sections after loading. Results demonstrate that regardless of the length change in the first or third section, the maximum value position moved towards the tip of the pile. The vertical stress is consequently transferred deeply, thereby avoiding extensive deformation in the shallow layer and ensuring the stability of the composite foundation. Additionally, nonlinearity in vertical stress variation was observed under different lengths of pile sections, highlighting the complexity of the load transfer mechanism.

**Figure 9:**
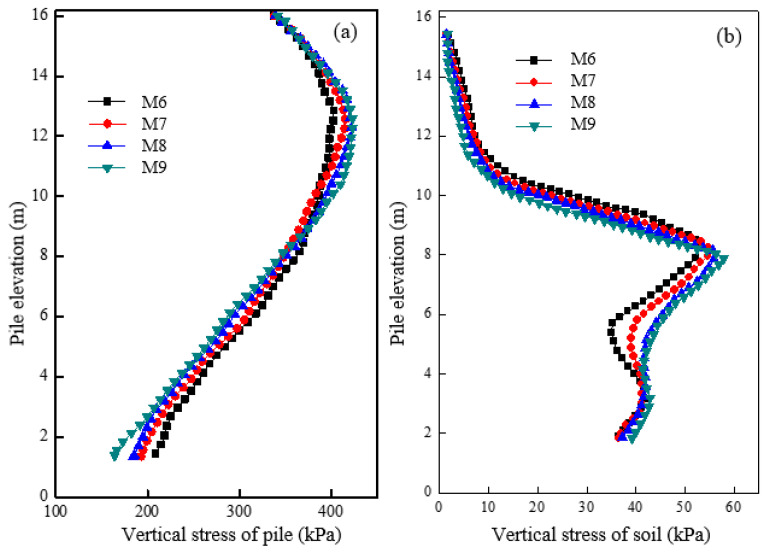
Variation of vertical stress under different concrete strengths of the first section in (**a**) pile and (**b**) soil.

Figure 9a shows the vertical stress distribution of piles with different concrete strengths after loading. Results show similar stress distribution even when the concrete pile section has different strengths. However, as the concrete strength increases, the vertical stress of the granular column section also slightly increases. The outcome implies that a higher concrete modulus results in increased load transfer efficacy and stress concentration. Figure 9b shows the vertical stress variation of soil at a distance of 0.15 m from the pile surface after loading. The change in the concrete strength caused a varying influence on the vertical stress of soil around the pile. With increased concrete strength, a decrease in the vertical stress of soil in the granular column section was observed, leading to an increase in the pile-soil stress ratio and deformation of the granular column.

**Figure 10:**
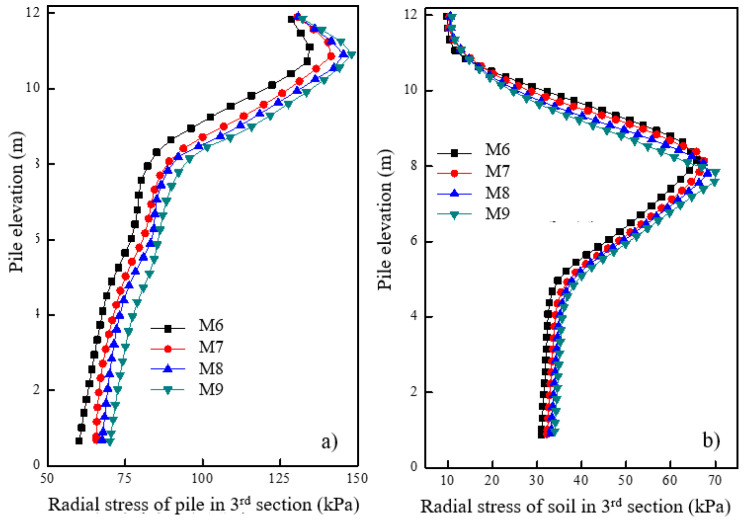
Variation of radial stress under different concrete strengths of the first section in (**a**) pile and (**b**) soil.

Figure 10a shows the pile’s radial stress variation in the granular column section under different concrete strengths after loading. As the concrete strength increased, the radial stress of the granular column also increased. Figure 10b shows the radial stress variation of soil at a distance of 0.15 m from the pile surface under different concrete strengths after loading. As the concrete strength increased, there was a small decrease in the radial stress of soil around the granular column. The increase in concrete strength results in the location of maximum radial deformation of the granular column transferring. Consequently, premature bulging deformation and partial bearing capacity loss are avoided.

**Figure 11:**
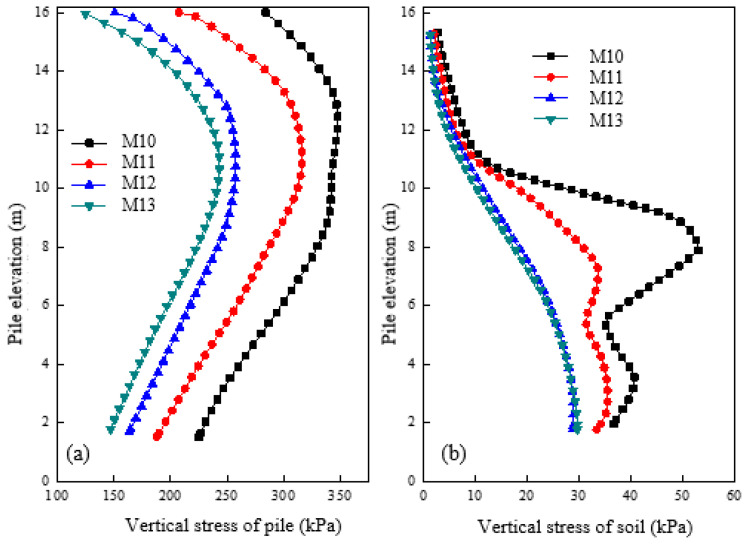
Variation of vertical stress under different pile diameters in (**a**) pile and (**b**) soil.

Figure 11a shows the vertical stress variation of piles under different pile diameters after loading. It can be observed that the vertical stress in M10 is nearly twice that of M12, while M12 has slightly less vertical stress compared to M13. As the pile diameter increases, the vertical stress of the pile gradually decreases. However, the reduction becomes less pronounced after reaching a critical diameter (referred to as a diameter of 1.2 m). Figure 11b shows the vertical stress variation of soil at a distance of 0.15 m away from the pile surface under different pile diameters after loading. The vertical stress of the soil around the pile decreases as the pile diameter increases, and significant differences can be observed between elevations of 6–11 m. The soil’s vertical stress gradually decreases as the pile diameter increases, although the values for M12 are nearly the same as in M13. It can be concluded that a critical diameter of 1.2 m is identified as the point where the vertical stress of both the pile and the surrounding soil increases.

**Figure 12:**
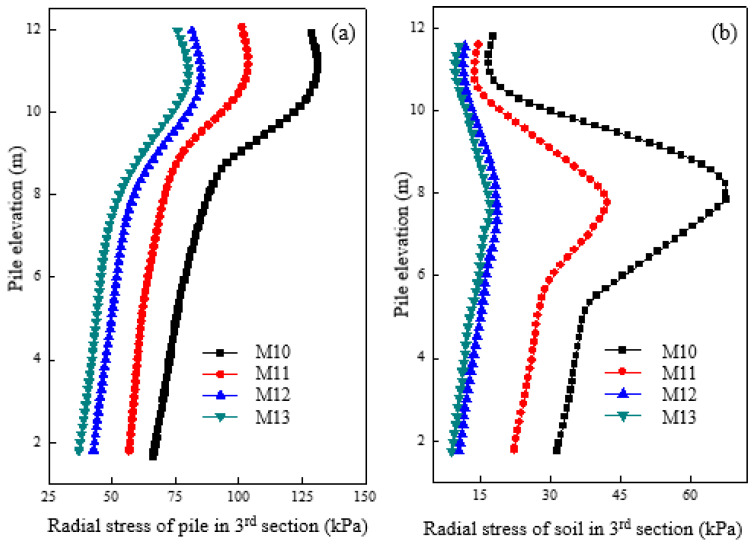
Variation of radial stress under different column diameters in (**a**) pile and (**b**) soil.

Figure 12a shows the radial stress variation of granular column under different pile diameters after loading. It was observed that an increase in pile diameter led to a decrease in the radial stress of the granular column. At the top of the column, a pile with a 0.6 m diameter had roughly double the radial stress compared to M11, whereas the stress level remained the same for columns with 1.2 m and 1.5 m diameters. Thus, increasing the diameter can reduce the radial stress of the granular column and prevent excessive deformation. Figure 12b shows the radial stress changes for soil located at 0.15 m from the pile surface under different pile diameters after loading. The radial stress of the soil decreased as the pile diameter increased. The results also suggested that there exists a critical column diameter of 1.2 m. When the column diameter was smaller than 1.2 m, increasing the column diameter could reduce the radial stress of the soil. Conversely, when the column diameter exceeded 1.2 m, the radial stress of the column and soil remained mostly unchanged.

**Figure 13:**
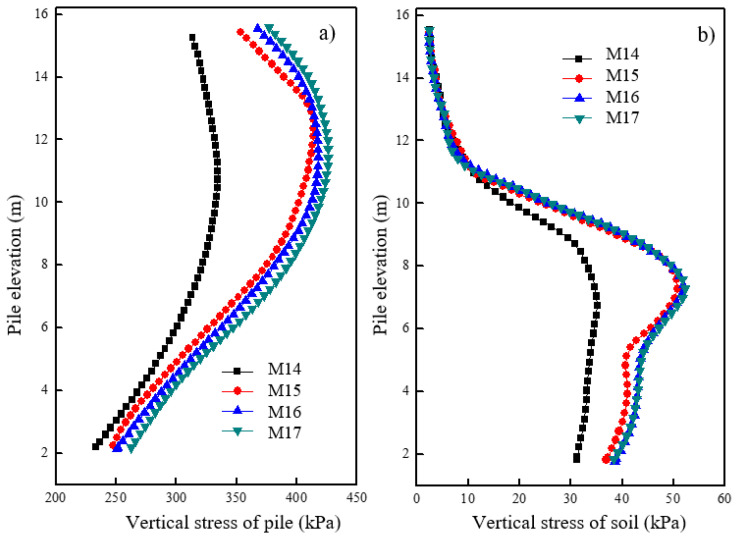
Variation of vertical stress under different pile spacings in (**a**) pile and (**b**) soil.

Figure 13a shows the variations in vertical stress for the pile at different pile spacings after loading. It was observed that smaller pile spacings resulted in lower vertical stress levels for the pile. Specifically, the vertical stress of a pile with a spacing of 1.2 m was considerably smaller than that with a pile space of 1.8 m. However, at pile spacings of 1.8 m, 2.4 m, and 3.0 m, there was no significant difference in the vertical stress levels observed. Figure 13b shows the vertical stress variation of soil at 0.15 m away from the pile surface under different pile spacings after loading. The vertical stress for soil in the first section remained almost the same across different pile spacings. The concrete pile section was capable of effectively transferring the load to the deeper parts, thereby decreasing the deformation in the shallow areas. However, a significant variation in vertical stress for the soil around the pile was observed in the granular column section at different pile spacings. Specifically, the vertical stress of soil around the granular column with a spacing of 1.2 m was significantly lower than that of a pile with a spacing of 1.8 m. No significant difference in the vertical stress of soil around the granular column was observed at pile spacings of 1.8 m, 2.4 m, and 3.0 m. Therefore, careful consideration of pile spacing should be given during the design process, and a spacing of 1.2 m may be appropriate.

**Figure 14:**
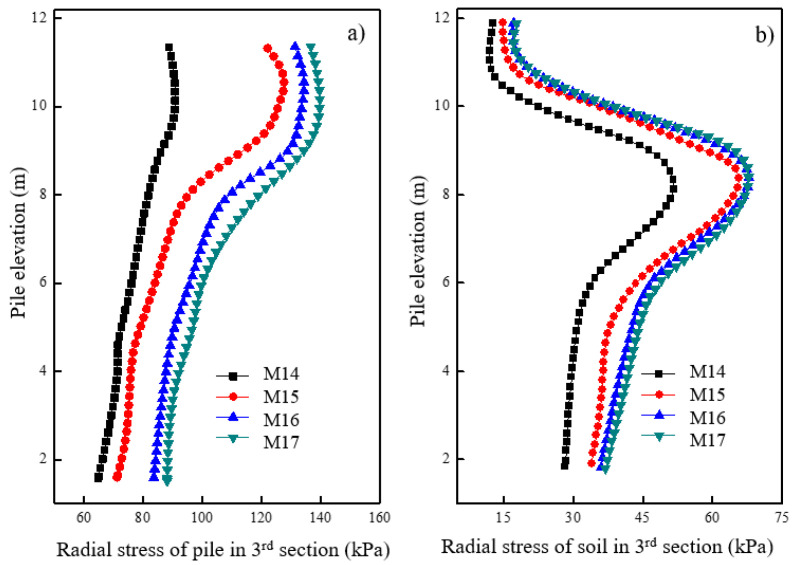
Variation of radial stress with different pile spaces in (**a**) pile and (**b**) soil.

Figure 14a shows the variations in radial stress for the granular column at different pile spacings after loading. It was observed that smaller pile spacings corresponded to lower radial stress levels for the granular column. Specifically, a significant reduction in radial stress for the granular column was observed when the pile spacing was 1.2 m. The radial stress variation for the granular column showed similar patterns when the pile spacing was reduced from 3.0 m to 2.4 m and from 2.4 m to 1.8 m. A pile spacing of 1.2 m resulted in a small radial stress, and no significant radial deformation was observed at the top of the column. Figure 14b shows the radial stress variation of soil at 0.15 m away from the pile surface under different pile spacings after loading. Smaller pile spacings corresponded to lower radial stress levels for the soil around the granular column. Specifically, the reduction in radial stress of soil around the granular column was significant when the pile spacing increased from 1.2 m to 1.8 m, and the distribution of radial stress in the soil around the granular column exhibited a similar pattern.

### 4.3. Effect of Soil Modulus

**Figure 15:**
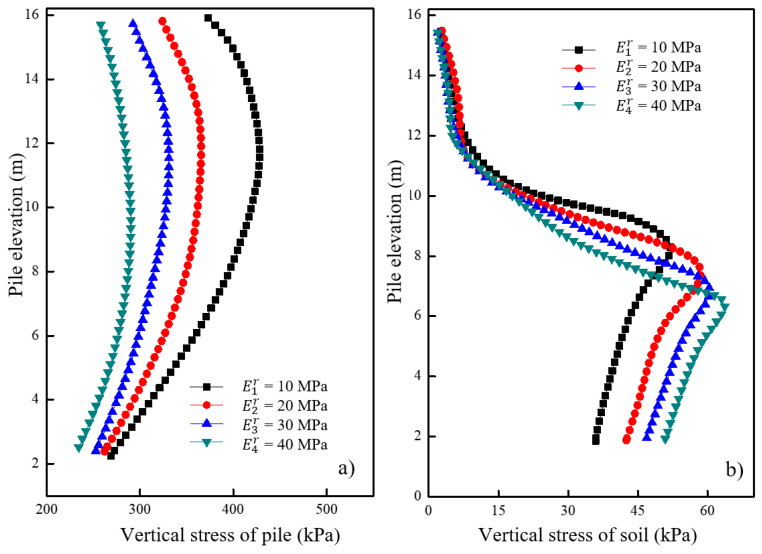
Variation of vertical stress under different moduli of reinforced soil in (**a**) pile and (**b**) soil.

Figure 15a shows the variations in vertical stress for the pile under different moduli of reinforced soil after loading. It was observed that the vertical stress of the pile decreased as the modulus of the reinforced soil increased. Furthermore, as the modulus of the reinforced soil increased, the reduction in vertical stress of the concrete pile section was more significant compared to the granular column section. Figure 15b shows the variations in vertical stress for the soil located 0.15 m away from the pile surface under different moduli of the reinforced soil after loading. The results showed that the greater the modulus of reinforced soil, the higher the vertical stress observed in the soil around the granular column. However, no significant change in vertical stress was observed around the soil in the concrete pile section. These findings suggest that a smaller ratio of pile-soil modulus leads to greater changes in the vertical stress of the pile and lower vertical stress in the soil.

**Figure 16:**
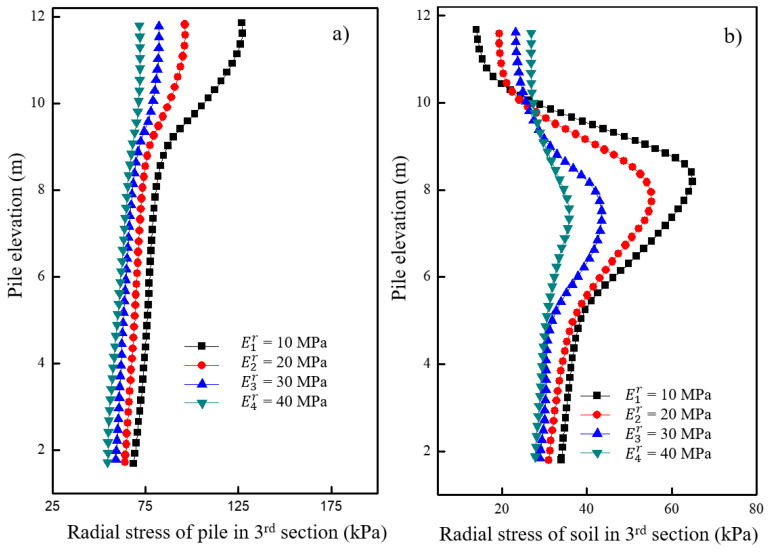
Variation of radial stress under different moduli of reinforced soil in (**a**) pile and (**b**) soil.

Figure 16a shows the variations in radial stress for the granular column under different moduli of reinforced soil after loading. It was observed that the radial stress of the granular column decreased as the modulus of the reinforced soil increased. The variation in radial stress at the top of the granular column was significantly greater than at the end of the granular column. As the modulus of reinforced soil increased, the proportion of the radial stress shared by the granular column decreased, reducing radial deformation and enabling the load to be transmitted deeper. Figure 16b shows the variations in radial stress for the soil located 0.15 m away from the pile surface under different moduli of the reinforced soil after loading. It was observed that a greater modulus of the reinforced soil resulted in a smaller decrease in radial stress for the soil located in the middle and at the end of the granular column. Conversely, the radial stress of the soil located around the top of the granular column had a slight increase. These findings suggest that increased modulus of the reinforced soil effectively constrained the granular materials, preventing significant bulging deformation and loss of bearing capacity.

**Figure 17:**
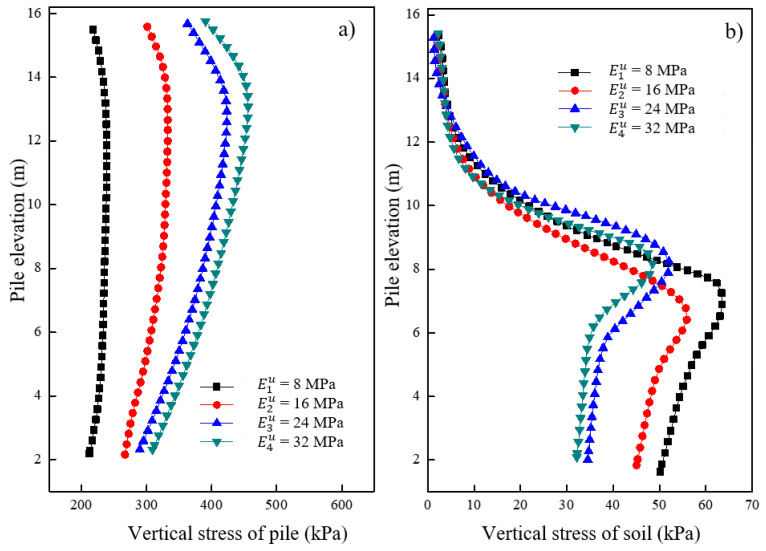
Variation of vertical stress under different moduli of underlying stratum in (**a**) pile and (**b**) soil.

Figure 17a shows the variations in vertical stress for the pile under different moduli of the underlying stratum after loading. It was observed that the vertical stress of the pile increased with an increase in the modulus of the underlying stratum. An increase in negative friction was observed in the concrete pile section with an increase in modulus. The effect of the underlying stratum’s modulus on the vertical stress of the pile decreased with an increasing modulus up to a certain value. Figure 17b shows the variations in vertical stress for the soil located 0.15 m away from the pile surface under different moduli of the underlying stratum after loading. The vertical stress of the soil around the granular column increased with a decrease in the modulus of the underlying stratum. However, no significant change in vertical stress was observed around the soil in the concrete pile section. The decrease in the modulus of the underlying stratum had a minimal effect on the vertical stress of soil in the concrete pile section but had a greater effect on the vertical stress of soil in the granular column section.

**Figure 18:**
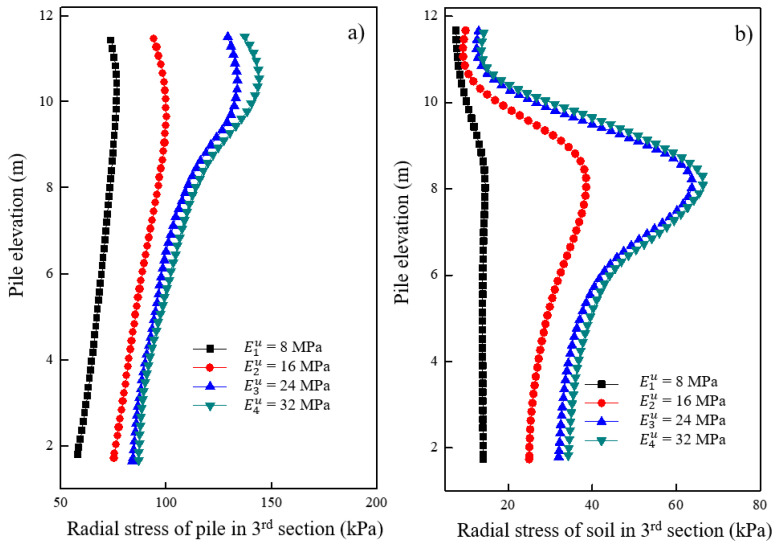
Variation of radial stress under different moduli of underlying stratum in (**a**) pile and (**b**) soil.

Figure 18a shows the variations in radial stress for the granular column under different moduli of the underlying stratum after loading. It was observed that the radial stress of the granular column increased with an increase in the modulus of the underlying stratum. Additionally, when the modulus of the underlying stratum increased from 24 MPa to 32 MPa, the increase in radial stress of the pile was significantly smaller than the situation when the modulus increased from 8 MPa to 16 MPa. The effect of the underlying stratum’s modulus on the radial stress of the granular column decreased up to a certain value. Figure 18b shows the variations in radial stress for the soil located 0.15 m away from the pile surface under different moduli of the underlying stratum after loading. A greater modulus of the underlying stratum resulted in a more significant increase in the soil’s radial stress around the elevation of 6–8 m. The increase in the underlying stratum’s modulus increased the radial deformation of the soil around the granular column. Similarly, the impact of the underlying stratum’s modulus on the soil’s radial stress around the granular column decreased to a certain value.

## 5. Limitations and Recommendations

According to the parametric study, the following recommendations of authors on the parameter selection were summarized in Table 5. The recommended values and their influence on the pile were extracted in detail, which could help to understand the behavior of the new type piles, improve the foundation clearly, and be convenient to design. This technique was expected to solve the bulging deformation of ordinary stone columns for weak foundation improvement. However, this paper was focused on the parametric study of the tandem compound pile’s improved foundation based on the continuum and discrete particles. The materials’ shape and fluid should be considered in the numerical analysis to model the real condition. The cushion, pile, and soil were selected as the variable to investigate the bearing capacity and deformation of the tandem compound piles’ improved foundation. The soil in the reinforced area might not be just a single layer, and the entire length of the pile is critical due to the pile possibly floating in the reinforced area. The above problems should be studied in the future.

## 6. Conclusions

In this study, a coupled continuum-discrete numerical method was developed to investigate the bearing capacity and deformation of the tandem compound piles’ improved foundation. The concrete pile section was represented as a continuous element using FLAC3D, while the granular column section was modeled as discrete particles using PFC3D. Parametric analysis was conducted by considering important factors such as cushion thickness and modulus, length, modulus, diameter, space of the tandem compound pile, and soil modulus. The key findings can be summarized as follows:

(1) In foundations without a cushion, the stress level of the pile was higher. However, utilizing a thick cushion did not fully exploit the bearing capacity of the pile. Therefore, it is necessary to determine an appropriate cushion thickness (e.g., 200–300 mm) and modulus (e.g., 90 MPa) to fully utilize the bearing capacity;
(2) Increasing the length and concrete strength of the concrete pile section, or modulus of the reinforced soil, effectively enhanced the bearing capacity. However, the impact of increasing the pile modulus on the bearing capacity was limited;
(3) The pile diameter inversely affected the vertical and radial stress of the pile, with larger diameters resulting in lower stress levels. However, the influence of pile diameter on the bearing capacity of the pile became insignificant once the pile diameter exceeded 1.2 m. The pile spacing should not be less than twice the pile diameter;
(4) A higher modulus of the underlying stratum resulted in increased vertical and radial stress in the pile, larger settlements on the top of the pile, and increased vertical deformation. Moreover, a critical modulus of the underlying stratum, 24 MPa, was observed in affecting the feature of pile and soil.

## Figures and Tables

**Table 1:** Particle size distribution of the granular column.

size/mm	60~40	40~20	20~10	10~5	5~2	2~1	1~0.5	<0.5
Percentage finer/%	60	20	10	5	3	2	0	0

**Table 2:** The properties of the tandem compound pile at different pile sections.

Pile Sections	Values	
Concrete section	*E*_1_ = 2.55 × 10^4^ MPa	*v*_1_ = 0.17
Middle section	*E*_2_ = 2.0 × 10^4^ MPa	*v*_2_ = 0.2
Granular column	$k_{n}$ = 6.0 × 10^4^ kN/m	$k_{n}^{b}$ = 130 N
	$k_{s}$ = 1.0 × 10^4^ kN/m	$k_{s}^{b}$ = 130 N

Notes: *E*_1_ and *E*_2_ are deformation modulus; *v*_1_ and *v*_2_ is Poisson ratio; $k_{n}$ is particle normal stiffness; $k_{s}$ is particle shear stiffness; $k_{n}^{b}$ is contact-bond normal stiffness; $k_{s}^{b}$ is contact-bond shear stiffness.

**Table 3:** The properties of the foundation soil.

Description	Unite Weight (kN/m^3^)	Elastic Modulus (MPa)	Poisson Ratio	Cohesion (kPa)	Friction Angle (°)
Cushion	21.8	30	0.30	0	45
Reinforced soil	17.0	10	0.35	12	10
Underlying stratum	20.5	24	0.30	30	26

**Table 4:** Parameter variation of different pile sections.

Pile Section	Length (m)					Concrete Strength				Pile Diameter (m)				Pile Spacing (m)			
Concrete section	2.5	5.0	7.5	2.5	2.5	C15	C20	C30	C40	0.6	0.9	1.2	1.5	1.2	1.8	2.4	3.0
Middle section	1.5	1.5	1.5	3.0	4.5												
Granular column	12	9.5	7	10.5	9												
Model description	M1	M2	M3	M4	M5	M6	M7	M8	M9	M10	M11	M12	M13	M14	M15	M16	M17

**Table 5:** Recommendation for parameter selection.

Description	Cushion		Pile Parameter				Soil Modulus	
	Thickness	Modulus	Length	Concrete Strength	Diameter	Spacing	Reinforced Area	Underlying Stratum
Recommended value	200–300 mm	90 MPa	-	-	1.2 m	1.8 m	-	24 MPa
Influence on vertical stress of pile	Decreasing 33.13%	Increasing 54.06%	-	Slightly	Decreasing 59.24%	Increasing 61.35%	Negative correlation	Increasing 220.88%

## Data Availability

Some or all data, models, or codes generated or used during the study are available from the corresponding author upon request.
